# Working memory in schizophrenia: The role of the locus coeruleus and its relation to functional brain networks

**DOI:** 10.1002/brb3.2130

**Published:** 2021-03-30

**Authors:** Stefanie Suttkus, Andy Schumann, Feliberto de la Cruz, Karl‐Jürgen Bär

**Affiliations:** ^1^ Lab for Autonomic Neuroscience, Imaging and Cognition (LANIC) Department of Psychosomatic Medicine and Psychotherapy University Hospital Jena Germany

**Keywords:** default mode network, executive control network, fMRI, locus coeruleus, schizophrenia, working memory

## Abstract

Evidence suggests functional brain networks, especially the executive control network (ECN) and default mode network (DMN), to be abnormal in schizophrenia. Dysfunctions within the locus coeruleus (LC)‐noradrenaline (NE) system, which is supposed to be pivotal to modulate neuronal network activation during executive control (e.g., working memory function), are also considered to play a vital role in the occurrence of positive (e.g., hallucinatory) or negative (e.g., inattentive) symptoms in these patients. In the present study, we sought to shed further light on the role of the LC‐NE system in patients with schizophrenia. More specifically, we wanted to improve our understanding of the relationship and possible disturbances of the ECN and DMN during a working memory task in patients. A total of 58 healthy control subjects and 40 medicated patients with schizophrenia were investigated using a working memory 3‐back task during functional magnetic resonance imaging. Main findings of our present study were differential dynamics of ECN and DMN blood oxygenation level‐dependent (BOLD) activations with increasing task demands in both patients and controls. Moreover, we found increased BOLD activation in the LC in patients compared to controls in the interaction contrast between groups and conditions. LC BOLD activation significantly correlated with both, the main hub of the ECN, that is, the dorsolateral prefrontal cortex, and of the DMN, that is, the posterior cingulate cortex. Thus, the LC‐NE system seems to be crucial in modulating neuronal network activity in a 3‐back working memory task and might significantly contribute to cognitive impairments in schizophrenia.

## Graphical abstract

In the present study, we sought to shed further light on the role of the LC‐NE system in patients with schizophrenia. We found the LC‐NE system to be crucial in modulating neuronal network activity in a 3‐back working memory task in schizophrenia.

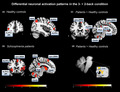

## 1 INTRODUCTION

*Working memory* (WM) is a crucial cognitive competence when multiple goals are pursued. In addition, it is important to guide behavior on the basis of information that is not available in the direct environment (Braver & Ruge, 2006; D'Esposito & Postle, 2015). As an important part of higher cognitive functions, working memory enables us to temporarily hold and manipulate information with limited capacity (Baddeley, 2012). The n‐back task is a popular working memory task which asks subjects to monitor a series of, for instance, verbal stimuli and to indicate when the currently presented stimulus is the same as the one presented n trials previously. Thus, online monitoring, updating, and manipulation of information are required (Owen et al., 2005).

Functional MRI studies consistently report a cortico‐striatal network subserving WM processes. This *executive control network* (ECN) encompasses core hubs such as the dorsolateral prefrontal cortex (DLPFC), ventrolateral prefrontal cortex (VLPFC), parietal cortex, and striatum (Murty et al., 2011; Tan et al., 2007). Working memory impairments and ECN dysfunction are consistently reported in psychiatric diseases such as schizophrenia or attention‐deficit hyperactivity disorder (Jiang et al., 2015; Kofler et al., 2018). For instance, Jiang et al., (2015) investigated how neuronal activation is altered in schizophrenia using a n‐back working memory task that has included a 0‐back and a 2‐back condition. They found patients compared to healthy controls showing an exaggerated response in the right DLPFC (Brodmann area (BA) 46) and bilateral VLPFC, as well as reduced activation in the bilateral DLPFC (BA 9). Jansma et al., (2004) used a parametric fMRI design and a spatial 3‐back working memory task to examine relations between working memory load, performance, and brain activity in patients with schizophrenia taking second‐generation antipsychotics compared to healthy controls. Despite the increasingly poor performance in patients, activity increased normally in DLPFC and inferior parietal cortex bilaterally as well as in the anterior cingulate cortex (ACC) with increasing working memory load. At the 3‐back condition, activity dropped in the DLPFC in comparison with controls, but not in the other regions. The results indicate that peak activation of the WM system is reached at a lower processing load in patients than in healthy controls. As a decline of DLPFC activity at high processing loads in itself is not abnormal, WM dysfunction in schizophrenia was suggested to be the result of an impaired functional output of the whole WM system.

Prior work has especially focused on the role of the ECN in working memory, but recent research suggests that the ECN is just one of several interacting networks being critical for task engagement (Kamp et al., 2018; McCormick & Telzer, 2018; Vatansever et al., 2015). Specifically, the *default mode network* (DMN) has gained attention in this respect. The DMN refers to a resting‐state network that is thought to show greater activity at rest. More specifically, the DMN tends to be active when one is engaging in internally driven cognitive processes such as self‐generated thoughts, mind‐wandering, or autobiographical memory retrieval (Andrews‐Hanna et al., 2014; Raichle et al., 2001). Core hubs of the DMN are the posterior cingulate cortex (PCC) and the adjacent precuneus (Prec), medial prefrontal cortex (mPFC), mesial and inferior temporal lobes (mTL/iTL), and inferior parietal lobe (iPL) (Bär et al., 2016; Raichle et al., 2001). In most cognitive control tasks, where attention is directed externally, the ECN shows increases and the DMN decreases in neuronal activation. Unsworth and Robison (2017) summarized that during attention control tasks, also including working memory tasks, lapses of attention and mind‐wandering are related to a reduced ECN and increased DMN BOLD activation. In consequence, goal‐directed behavior often worsens. Thus, in many cognitive control tasks where attention has to be allocated to external stimuli interactions between the ECN and DMN are critical for success. The ECN is needed to maintain task goals and prevent lapses of attention by suppressing the DMN.

In patients with schizophrenia, many fMRI studies have demonstrated abnormal activity within the DMN during a broad range of tasks (Hu et al., 2017), also including working memory tasks (Kim et al., 2009; Pomarol‐Clotet et al., 2008). Reduced suppression of the DMN in patients with schizophrenia is often interpreted as a failure to allocate cognitive resources adequately resulting in an impaired task performance. However, whether reduced suppression of the DMN is better interpreted as the cause or the consequence of impaired cognition is still a matter of debate. Interestingly, in a study by Whitfield‐Gabrieli et al., (2009) patients with schizophrenia were found to continue to exhibit reduced DMN suppression, especially in the MPFC, even when statistically controlling for cognitive performance as well as when analyzing an easy task condition in which patients performed comparable to controls.

One major candidate influencing PFC function in schizophrenia is the norepinephrine (NE) system which arises from the locus coeruleus (LC) in the brainstem. The LC‐NE system widely projects throughout the cortex and is well suited to modulate widely distributed neuronal networks such as those engaged by the PFC during higher‐order cognition. There is plentiful evidence in both animal and human studies that NE strongly modulates PFC function during cognitive processes, that is, working memory (Aston‐Jones & Cohen, 2005; Durstewitz & Seamans, 2008; Moore et al., 1999; Robbins & Arnsten, 2009). In a recent network analysis, we found evidence that the LC is integrated into the ECN (Bär et al., 2016).

The importance of the LC‐NE system for working memory and attention is also emphasized by psychopharmacological studies. For example, it has been shown that drugs increasing the central NE concentration (e.g., modafinil) lead to more subjective alertness and a better performance on some attention control and working memory measures (Chamberlain & Robbins, 2013). Modafinil has also been shown to be related to the deactivation of the DMN during task performance (Minzenberg et al., 2018). Furthermore, pharmacological manipulations typically depend on baseline levels of arousal, suggesting the importance of tonic NE levels in determining the attentional state (Coull et al., 2004; Smith & Nutt, 1996).

While a major prevailing hypothesis is that altered dopaminergic and/or glutamatergic signaling contributes to the development and etiology of schizophrenia, there is also evidence that the LC‐NE system might be involved (Borodovitsyna et al., 2017; Yamamoto & Hornykiewicz, 2004). It has been proposed that the development of both positive (delusions, hallucinations, and thought disorder) and negative symptoms (affective blunting, inattention, and abulia) of schizophrenia might be related to NE dysregulation. For instance, Yamamoto and Hornykiewicz (2004) concluded that the psychopathology of positive and negative symptoms might be caused from hyper‐ and hypo‐vigilant states of consciousness, respectively. Other imaging studies using positron emissions tomography also proposed hyper‐activation of the temporal cortex and limbic areas, as well as hypo‐activation of prefrontal areas as correlates of positive and negative symptoms, respectively (Andreasen et al., 1992; Silbersweig et al., 1995). Further, NE has been found to be elevated in both the blood plasma (Kemali et al., 1982) and cerebrospinal fluid of patients with schizophrenia, especially those with positive symptoms (Kemali et al., 1990; Lake et al., 1980). Postmortem studies have also reported increased markers for NE in the brains of patients who suffered from schizophrenia (Bird et al., 1980; Farley et al., 1978).

### 1.1 Objectives and hypotheses

In the present study, we want to shed further light on the role of the LC‐NE system in patients with schizophrenia being a key neurotransmitter system to modulate neuronal network activation. More specifically, we want to improve our understanding of the relationship and possible disturbances of human brain networks during a working memory task in patients. ***First***, considering previous findings, we assume a reduced working memory performance in schizophrenia patients compared to healthy controls. Our ***second*** assumption is that core regions of the ECN, especially the DLPFC, show less BOLD activation during working memory performance in SZ patients compared to healthy controls. ***Third***, we also hypothesize less deactivation of DMN core nodes such as the PCC in SZ patients compared to HC especially when cognitive demand is highest. ***Lastly***, we suppose that LC BOLD activation is positively related to DLPFC and negatively related to PCC BOLD activations in HC, but not in patients.

## 2 METHODS

### 2.1 Sample characteristics

A total of 58 healthy control subjects (40 male and 18 female) were recruited by local newspaper advertisement and screened for psychiatric or neurological diseases by a psychiatrist. Subjects with past or current neurological or psychiatric diseases and/or first‐degree relatives with axis I psychiatric disorders were excluded from the study. 40 patients (29 male and 11 female) meeting the DSM‐IV criteria for schizophrenia according to the Structured Clinical Interview (SCID) for DSM‐IV Axis I disorders were recruited and screened by a psychiatrist from the inpatient service of the psychiatric university hospital in Jena. Patients were included in the study during symptom remission and not in an acute state of the disease. Patients with a current comorbid axis I disorder (according to SCID) or with neurological disorders were excluded from this study. 39 patients received second‐generation antipsychotics (see Table S1). The antipsychotic treatment was quantified using chlorpromazine (CPZ) equivalents (Andreasen et al., 2010). The mean CPZ equivalent was 652.55 (*SD* = 460.45) mg/day. One patient was medication naïve. Six patients were additionally treated with a selective serotonin reuptake inhibitor (SSRI; Sertralin, Escitalopram). The patients' psychopathological status was assessed using the Scales of Assessment of Positive and Negative Symptoms (SAPS and SANS). Patients' scores were *M* = 23.49 (*SD* = 17.82) on SAPS and *M* = 19.38 (*SD* = 16.82) on SANS. The mean age at onset of schizophrenia was 26.52 years (*SD* = 8.38). On average, patients reported an illness duration of *M* = 7.85 (*SD* = 8.64) years.

In healthy controls, the mean age was 34.47 (*SD* = 12.56) years. Patients had a mean age of 35.3 (*SD* = 12.7) years. Duration of school education was 11.43 (*SD* = 0.99) years for HC and 10.95 (*SD* = 1.29) years for patients. A two‐sample *t* test showed no significant group differences regarding age (*t*(96)=−0.32, n.s.), no significant difference in education (*t*(64.83)=1.96, *p* =.06), and no significant differences in overall impulsivity (BIS; *t*(54.94)=−1.95, *p* =.06). We found significant group differences in trait (STAI trait; *t*(50.28)=−6.41, *p* <.001) and state anxiety (STAI state; *t*(49.9)=−4.6, *p* <.001) as well as inferential logic (LPS; *t*(68.99)=3.77, *p* <.001). However, in both HC and SZ inferential logic is within the average range. Sample characteristics are summarized in Table 1.

**TABLE 1:** Demographic and clinical data of healthy controls and patients

	Healthy controls *M* (*SD*)	SZ patients *M* (*SD*)	Group difference *p* value
Gender (m/f)	40/18	29/11	‐
Age (in years)	34.47 (12.56)	35.3 (12.7)	*n*.s.
Education (in years)	11.43 (0.99)	10.95 (1.29)	*n*.s.
Duration of illness (in years)	*n*.a.	7.85 (8.64)	‐
Age of onset (in years)	*n*.a.	26.52 (8.38)	‐
SAPS	*n*.a.	23.49 (17.82)	‐
SANS	*n*.a.	19.38 (16.82)	‐
BIS−11	59.66 (8.67)	64.39 (12.83)	*n*.s.
STAI state	32.33 (6.72)	42.46 (12.29)	*p* <.001
STAI trait	32.74 (6.74)	47.34 (12.95)	*p* <.001
LPS	114.79 (10.96)	104.59 (11.82)	*p* <.001

Abbreviations: SZ – schizophrenia, SAPS/SANS – Scales of Assessment of Positive and Negative Symptoms, BIS‐11 – Barrat Impulsivity Scale, STAI state/trait – State‐Trait Anxiety Inventory, LPS – Performance Testing System, m – male, f – female, *n*.a. – not available, *n*.s. – nonsignificant, *M* – mean value, *SD* – standard deviation.

All subjects were German native speakers, right‐handed according to the modified version of Annetts Handedness Inventory (Briggs & Nebes, 1975), and provided written informed consent prior to participating in the study. The study protocol was approved by the Ethics Committee of the University of Jena. All subjects were paid 8 Euro per hour for their participation.

### 2.2 Experimental design

We used a parametric n‐back task containing a baseline condition (x‐back) and three different load levels (1‐back, 2‐back, and 3‐back). The working memory 3‐back task was performed during a functional MRI scan and was arranged as a block design with an overall number of five blocks of each condition. Using the Presentation software package (Neurobehavioral Systems Inc., USA), single white letters (A–Z) were presented in a pseudo‐randomized order and appeared for 1800ms on a black background. All letters were separated by an interstimulus interval (blank screen) lasting 2500ms. During the baseline condition, participants had to react by button press as soon as an “X” appeared on the screen. The 1‐back condition demanded a response to any letter that matched the last letter seen. The 2‐back condition demanded a response to any letter that matched the last but one letter seen. In the 3‐back condition, participants were required to respond to any letter that matched the last but two letters. Each task block started with the presentation of the following task condition and was presented for 4000ms. The instruction was followed by a fixation cross which appeared for another 4000ms before the presentation of the single letters. Task conditions were presented in a pseudo‐randomized order. Letters were presented with a ratio of 85 nontargets to 25 targets in each condition.

Visual stimuli were projected on to a transparent screen inside the scanner tunnel which could be viewed by the subject through a mirror system mounted on top of the MRI head coil. The subjects’ responses were registered by an MRI‐compatible fiber optic response device (Lightwave Medical Industries, Canada) with one response button on a keypad for the right hand.

### 2.3 Assessment scales

To better describe sample characteristics, we collected different scales. Impulsivity, as a personality trait, was assessed by the Barratt Impulsivity Scale 11 (BIS‐11; Patton et al., 1995). The purpose of the State‐Trait Anxiety Inventory (STAI; Laux et al., 1981) is to measure the presence and severity of current symptoms of anxiety as well as a general tendency to be anxious. Inferential logic was assessed using a subtest of the Performance Testing System (LPS; Horn, 1983) to get an idea of current cognitive abilities of the participants. The patients' psychopathological status was assessed using the Scales of Assessment of Positive and Negative Symptoms (SAPS and SANS).

### 2.4 Data acquisition

Data were collected on a 3T whole‐body system equipped with a 64‐element receive‐only head matrix coil. T2*‐weighted images were obtained using a gradient‐echo EPI sequence (TR = 2120ms, TE = 36ms, TA = 2100ms, FOV = 224mm^2^, acquisition matrix = 160×160 mm^2^, and flip angle = 90°) with 104 interleaved transverse slices of 1.4 mm thickness, a multi‐band acceleration factor of 4, and with an in‐plane resolution of 1.4 × 1.4 mm^2^. A series of 626 whole‐brain volume sets were acquired in one session lasting approximately 25 min. High‐resolution anatomical T1‐weighted volume scans (MP‐RAGE) were obtained in sagittal orientation (TR = 2300ms, TE = 3.03ms, TI = 900ms, flip angle = 9°, FOV = 256mm×256mm, matrix 256 × 256, number of sagittal slices = 192, and acceleration factor (PAT = 2) with an isotropic resolution of (1 × 1×1) mm^3^).

### 2.5 Physiological recordings during fMRI

During the fMRI scan, respiratory and cardiac signals were recorded simultaneously using an MR‐compatible BIOPAC MP150 polygraph (BIOPAC Systems Inc., Goleta, CA, USA) and digitized at 500 Hz. Respiratory activity was assessed by a strain gauge transducer incorporated in a belt tied around the chest, approximately at the level of the processus xiphoideus. The cardiac signal, photoplethysmograph (PPG) signal, was recorded using a pulse oximeter attached to the proximal phalanx of the index finger of the subject's left hand.

To remove MRI‐related or movement artifacts, the PPG signal was band‐pass filtered (0.05–3 Hz), and the respiratory signal was low‐pass filtered with a cutoff frequency of 10 Hz. Pulse‐wave onsets were automatically extracted by detecting peaks of the temporal derivative of the filtered PPG signal (Schumann et al., 2018). The quality of peak detection was visually inspected by an expert and corrected when necessary.

### 2.6 fMRI preprocessing

Data analysis was performed using SPM12 (http://www.fil.ion.ucl.ac.uk/spm) and AFNI software package (https://afni.nimh.nih.gov/). The first four images were discarded to ensure a steady‐state tissue magnetization condition. Time‐locked cardiac and respiratory artifacts as well as slow blood oxygenation level fluctuations were removed using RETROICOR (Glover et al., 2000) and respiration volumes per time regressors (Birn et al., 2008). RETROICOR and RVT regressors were generated on a slice‐wise basis by AFNI's “RetroTS.m” script (Jo et al., 2010).

Further preprocessing steps of the fMRI data included slice timing correction, rigid body realignment to the mean of all images, and alignment of functional and anatomical data. Afterward, images were normalized to the MNI space using the DARTEL procedure integrated into SPM12 (Ashburner, 2007) and smoothed with a Gaussian kernel of 6 mm full width at half‐maximum.

To accurately identify nuclei within the midbrain and brainstem for the subsequent time‐series extraction, neuroimaging data were normalized to the spatially unbiased infra‐tentorial template (SUIT, version 3.1; Diedrichsen, 2006). This procedure was performed after following all previous steps up to coregistration. Using the SUIT toolbox, we applied the following preprocessing steps: (i) segmentation of the whole‐brain image dataset as implemented in SPM12, (ii) cropping of the image dataset, retaining only the cerebellum and brainstem, (iii) normalization using the DARTEL engine (Ashburner, 2007), which uses gray‐ and white‐matter segmentation maps produced during cerebellar isolation to generate a flowfield using Large Deformation Diffeomorphic Metric Mapping (LDDMM; Miller et al., 2005), and (iv) reslicing to a voxel size of (1.5 × 1.5 × 1.5) mm^3^.

### 2.7 fMRI data analysis

Performance was assessed by the number of correct reactions in each condition. Repeated measures ANOVAs with the within‐subject factor task (x‐back, 1‐back, 2‐back, 3‐back) and the between‐subject factor group (patients, controls) were performed.

Using the SPM12 software, a fixed‐effects model with a block design including both correct and false responses at a single‐subject level was performed to create contrast images of parameter estimates. Importantly, one regressor per condition was also included in modulating performance accuracy and, thus, controlling for performance. For each subject, the baseline condition (x‐back) was subtracted from the activation contrasts (1‐back, 2‐back, 3‐back). Thus, all comparisons were standardized on the control condition (x‐back). Final contrast estimates were then entered into a second‐level analysis. At the second level, a random‐effects full‐factorial design was used to investigate neuronal activation in the groups (i.e., patients and controls) and conditions (1‐back, 2‐back, 3‐back). A one‐way ANOVA with group (patients versus. controls) as the between‐subject factor was performed for each load condition (1‐back, 2‐back, and 3‐back). Post hoc, we also investigated potential associations between neuronal activation in our regions of interest, the dACC, PCC, and LC, by extracting and correlating parameter estimates. To this aim, ROIs were drawn around the voxel with maximum activation including all voxels within a 6mm radius. Beta values were then extracted from all voxels within these ROIs, and the first eigenvariate was calculated via singular value decomposition (SVD) and used for further data processing. If not indicated otherwise, within‐ and between‐group analyses were based on a voxel‐based threshold of 0.001 (uncorrected) and were false discovery rate (FDR) cluster corrected.

### 2.8 Behavioral data analysis

Behavioral data analyses were performed using SPSS Statistics V22. We conducted a repeated measures ANOVA with the within‐subject factor task (x‐back, 1‐back, 2‐back, and 3‐back) and the between‐subject factor group (healthy controls, HC; patients with schizophrenia, SZ). Post hoc *t* tests were used to test for between‐group differences in performance. To account for the problem of multiple comparisons, we adjusted the statistical significance level using the FDR approach.

### 2.9 Brainstem analysis

For the statistical comparison (ANOVA) based on our initial hypotheses, that is, regarding the activation differences in the LC between patients and controls, we used the small volume correction (SVC) method to account for the small size of the brainstem/midbrain nuclei. The LC was a priori anatomically defined as region of interests (ROIs). To obtain the anatomically most precise ROIs, we used the LC mask image in the MNI coordinate space based on Keren et al., (2009), which represents the extent of peak LC signal distribution, obtained from a sample of 44 healthy adults using high‐resolution T1‐weighted Turbo Spin Echo MRI. The statistical significance was set to *p* <.05, FWE voxel‐level corrected.

### 2.10 Post hoc subgroup analyses

As we expect that the most demanding condition has a strong influence on neuronal activation and performance, we divided two patient subgroups and performed post hoc subgroup analyses to get an even better understanding of the data at hand. We used the median split‐half method to generate a low‐ and a high‐performing patient group. The critical value calculated, using the performance in the 3‐back condition as reference, was 68% correct trials, and thus, all subjects with equal or below 68% accuracy were identified as low‐performing group and the subjects above 68% accuracy as high‐performing patient group.

In the patient group, we got *N* = 21 subjects in the low and *N* = 19 subjects in the high‐performing group. Sample characteristics of the patient subgroups are summarized in Table 2. To compare schizophrenia patients and healthy individuals more accurately on the neuronal level, we sought to divide the group of healthy individuals based on the performance of patients. However, only four healthy individuals achieved a performance comparable to the low‐performing patient group. Thus, it was not reasonable to divide healthy individuals based on the calculated accuracy cutoff. Consequently, we did not divide healthy subjects into subgroups. Nevertheless, we investigated whether the differences between patients and healthy individuals were caused by performance variations in the patient group. Thus, at first we compared the low‐ and high‐performing patients in the most demanding working memory condition. Accordingly, we then compared low‐ as well as high‐performing patients with the control subject group.

**TABLE 2:** Characteristics of the patient subgroups

	SZ *high* *M* (*SD*)	SZ *low* *M* (*SD*)	Group difference *p* value
Gender (m/f)	11/8	18/3	‐
Age (in years)	31.89 (10.17)	38.38 (14.15)	*n*.s.
Education (in years)	11.53 (1.07)	10.48 (1.3)	*p* =.011
Duration of illness (in years)	7.00 (6.47)	8.56 (10.23)	*n*.s.
Age of onset (in years)	24.87 (8.28)	27.89 (8.45)	*n*.s.
SAPS	21.83 (19.26)	25.05 (16.73)	*n*.s.
*Hallucinations*	4.67 (6.07)	3.58 (3.91)	*n*.s.
*Delusions*	9.72 (9.56)	8.32 (7.71)	*n*.s.
*Bizarre behavior*	0.72 (1.49)	2.00 (3.57)	*n*.s.
*Formal thought disorders*	6.72 (9.22)	10.21 (8.96)	*n*.s.
SANS	20.56 (15.15)	18.26 (18.61)	*n*.s.
*Affective blunting*	7.72 (8.84)	5.95 (8.08)	*n*.s.
*Alogy*	1.5 (2.79)	2.11 (3.97)	*n*.s.
*Aboulia and apathy*	2.72 (2.54)	2.74 (3.6)	*n*.s.
*Anhedonia*	8.39 (6.22)	6.63 (6.83)	*n*.s.
*Attention*	0.22 (0.94)	0.84 (1.68)	*n*.s.
CPZ (in mg)	628.67 (509.9)	672.44 (429.01)	*n*.s.
BIS−11	61.56 (9.93)	67.22 (14.94)	*n*.s.
STAI state	44.53 (11.2)	40.7 (13.16)	*n*.s.
STAI trait	49.56 (12.74)	45.35 (13.13)	*n*.s.
LPS	110.00 (10.71)	99.47 (10.69)	*p* =.005

Abbreviations: SZ – schizophrenia, SAPS/SANS – Scales of Assessment of Positive and Negative Symptoms, BIS‐11 – Barrat Impulsivity Scale, STAI state/trait – State‐Trait Anxiety Inventory, LPS – Performance Testing System, m – male, f – female, *n*.a. – not available, *n*.s. – nonsignificant, M – mean value, *SD* – standard deviation.

## 3 RESULTS

### 3.1 Behavioral results and task performance

The repeated measures ANOVA of the number of correct reactions with the within‐subject factor task (x‐back, 1‐back, 2‐back, 3‐back) and the between‐subject factor group (healthy controls, HC; patients with schizophrenia, SZ) revealed a significant main effect of task (*F*(1.917, 183.987)=83.288, *p* <.001; partial η^2^=0.465), a significant main effect of group (*F*(1, 96)=35.098, *p* <.001; partial η^2^=0.268), and a significant interaction between task and group (*F*(1.917, 183.987)=28.45, *p* <.001; partial η^2^=0.229).

**FIGURE 1:**
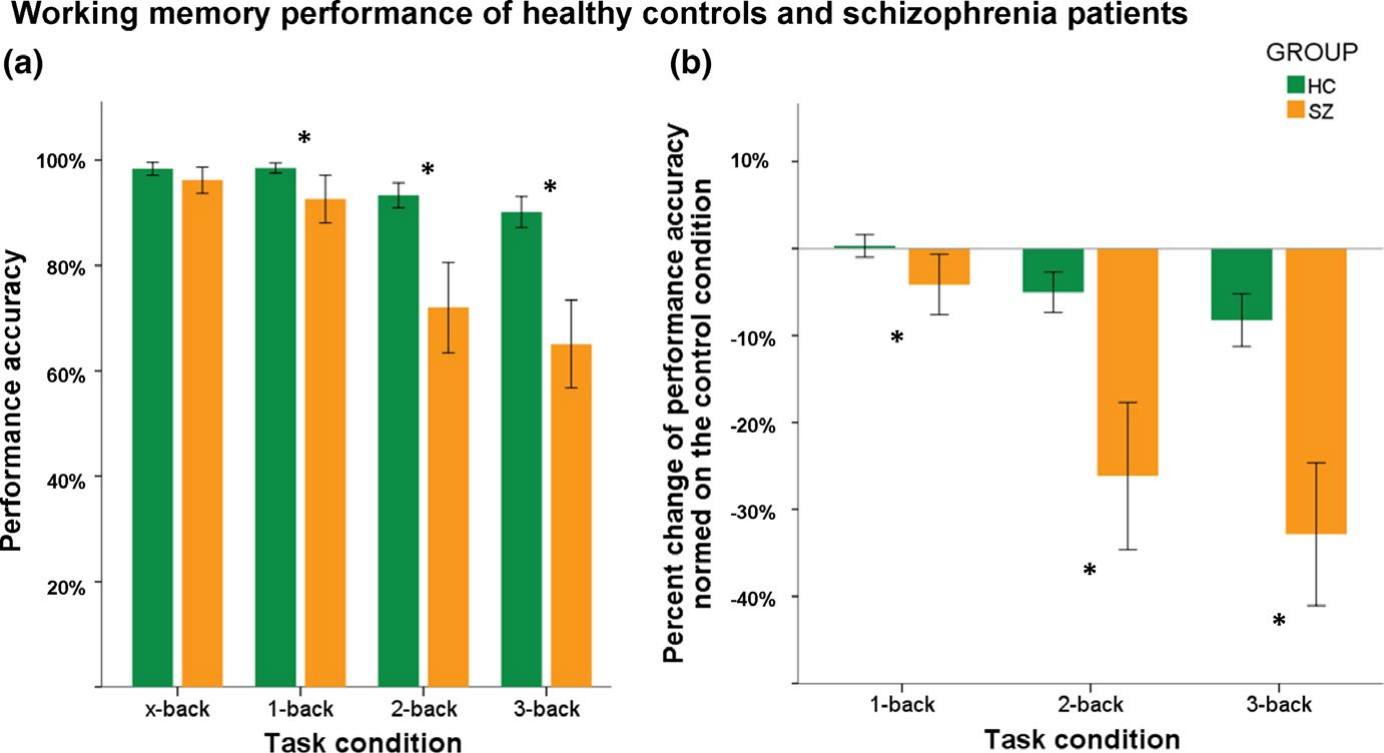
*A:* Performance accuracy in both groups for all task conditions; *B:* Percent change of performance accuracy standardized on the control condition (x‐back) for healthy controls and patients. *Abbreviations:* HC – healthy controls (green), SZ – patients with schizophrenia (orange); * ‐ significant group difference (*p* <.05)

As indicated by post hoc *t* tests, patients performed significantly worse in the 1‐, 2‐, and 3‐back condition compared to healthy controls (1‐back: *t*(42.413)=2.556, *p_FDR_* = 0.019, 2‐back: *t*(45.116)=4.842, *p_FDR_ = *0.002, 3‐back: *t*(48.969)=5.738, *p_FDR_ = *0.002). There was no significant group difference in the control condition (x‐back: *t*(57.417)=1.566, *p* =.123). Figure 1 pictures performance accuracy in both groups for all task conditions (Figure 1A) on the left‐handed side. On the right‐handed side, the percent change of performance accuracy normed on the control condition for healthy controls and patients is pictured (Figure 1B).

### 3.2 Differential neuronal (de‐)activation patterns within and between groups

**FIGURE 2:**
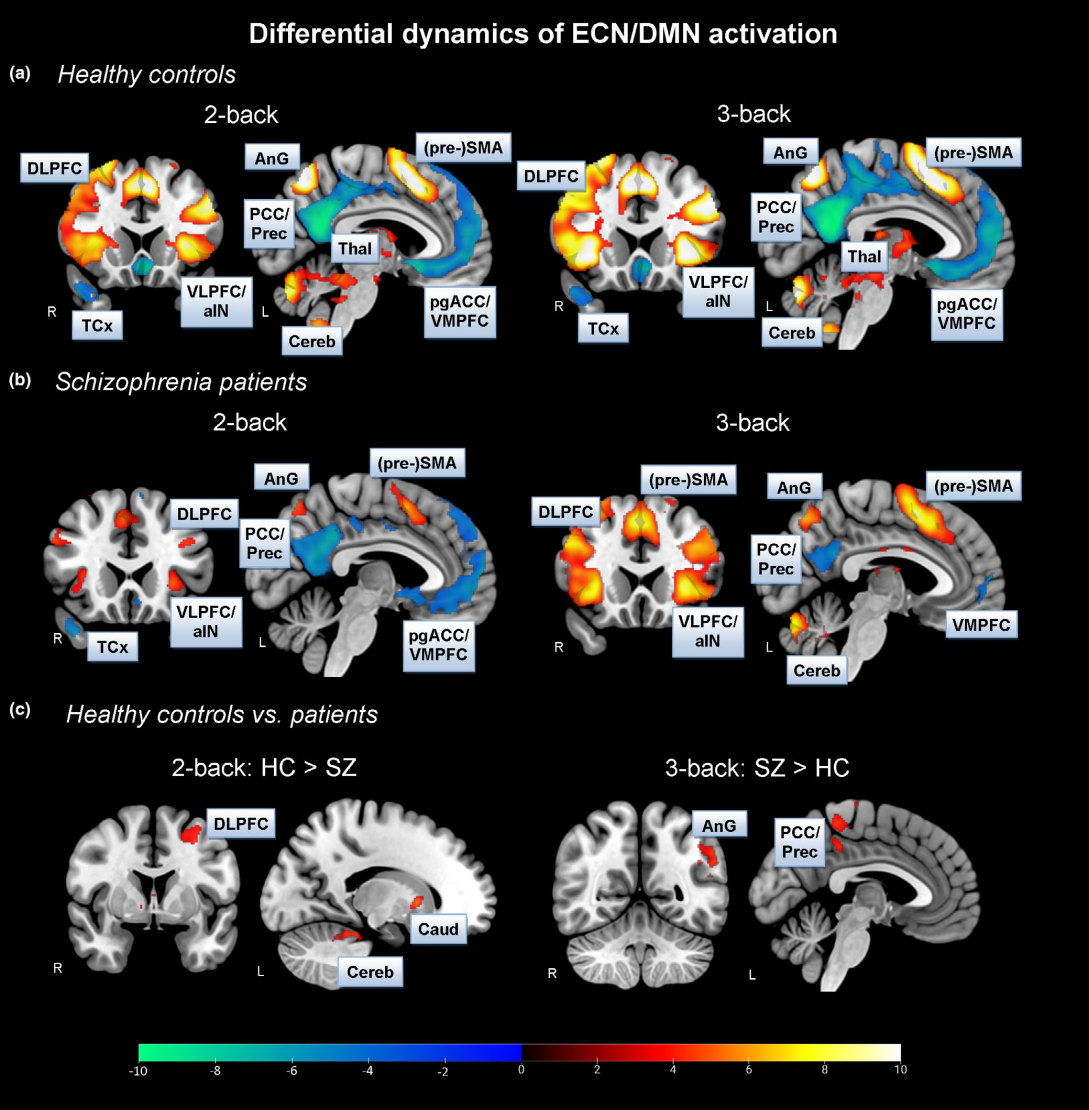
*Executive Control Network activation (yellow‐red color) and Default Mode Network deactivation (blue‐green color). Within‐ and between‐group differences*: All whole‐brain analyses were performed using a voxel‐level of *p* <.001 (uncorr.) and cluster level of *p* <.05 (FDR corr.). See positive BOLD activation changes in yellow‐red color and negative BOLD activation changes in blue‐green color. A – Neuronal activation *(yellow‐red color)* and deactivation *(blue‐green color)* patterns in the 2‐ and 3‐back condition in healthy controls. B – Neuronal activation *(yellow‐red color)* and deactivation *(blue‐green color)* patterns in the 2‐ and 3‐back condition in patients with schizophrenia. C – Neuronal activation patterns in the 2‐ and 3‐back condition between healthy controls and patients. Abbreviations: SZ – schizophrenia, HC – healthy controls, PCC – posterior cingulate cortex, Prec – precuneus, AnG – Angular Gyrus, pgACC – perigenual anterior cingulate cortex, DLPFC – dorsolateral prefrontal cortex, VLPFC – ventrolateral prefrontal cortex, VMPFC – ventromedial prefrontal cortex, aIN – anterior insula, (pre‐)SMA – (pre)supplementary motor area, TCx – temporal cortex, Thal – thalamic nucleus, Cereb – cerebellum, R – right hemisphere, L – left hemisphere

In general, we found increased BOLD activation patterns during 2‐ and 3‐back in core hubs of the ECN, especially the DLPFC and VLPFC, in both healthy controls and schizophrenia patients (Figure 2A&B (yellow‐red color); Table S2, S3). Compared to x‐back, we were also able to identify BOLD deactivations in core hubs of the DMN, that is, the PCC and VMPFC, in HC as well as in patients (Figure 2A&B (blue‐green color); Table S2, S3).

As hypothesized, we found significantly increased BOLD activations in, for instance, the DLPFC, in the 2‐back condition in HC compared to SZ patients (Figure 2c; Table S4). Surprisingly and in contrast to our expectation, we did not find differences in DLPFC activation between groups in the most demanding 3‐back condition. However, in the 3‐back contrast, we identified significantly increased BOLD activation patterns in regions associated with the DMN, especially the PCC, in patients compared to HC (Figure 2c; Table S4). This finding, however, met our expectation. There were no significant BOLD activation differences in the easy 1‐back condition between both groups.

### 3.3 Neuronal activation patterns with regard to the transition from the 2‐ to 3‐back condition

**FIGURE 3:**
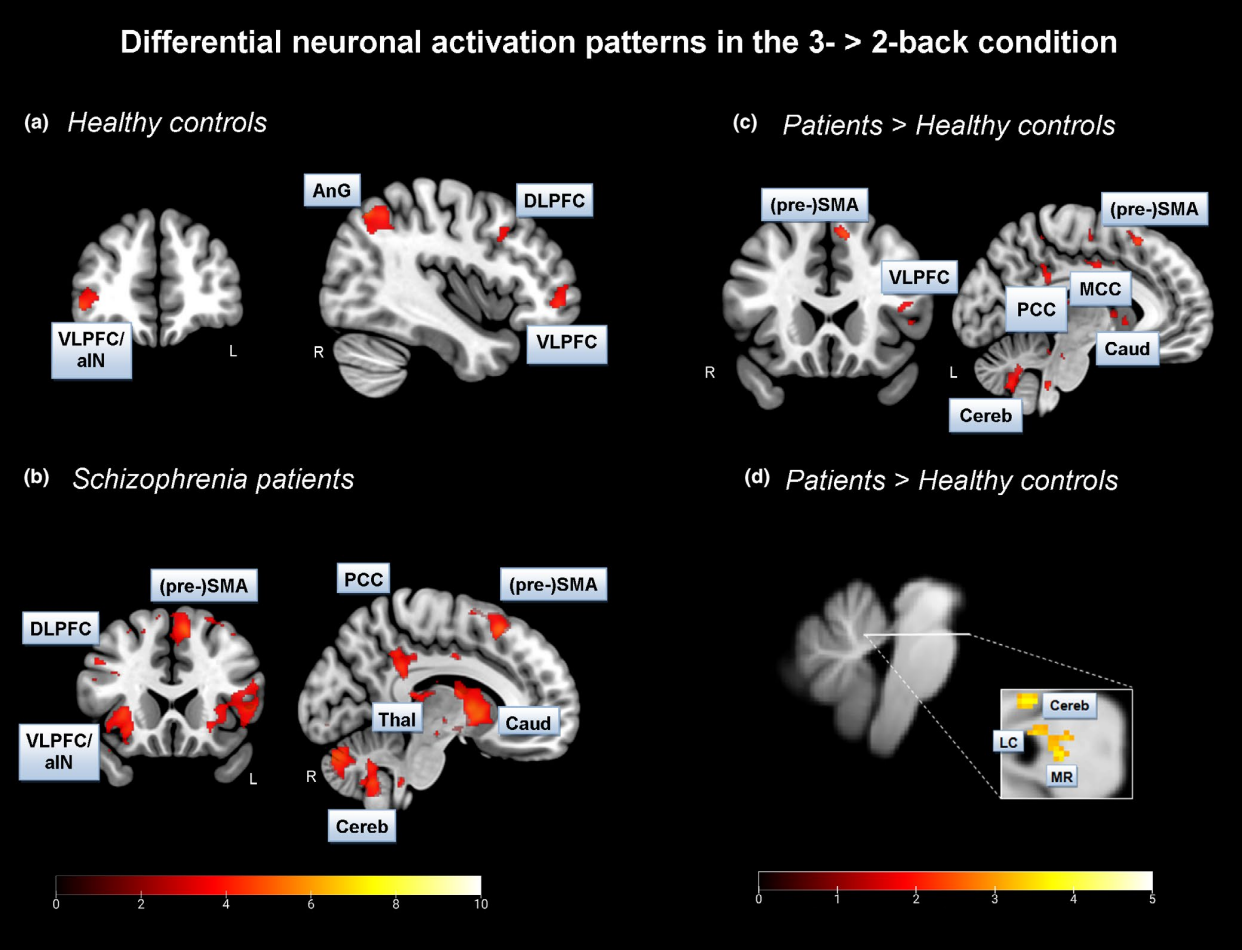
*Differential neuronal activation patterns in the 3‐ > 2‐back contrast of the n‐back task*: Whole‐brain analyses were performed using a voxel‐level of *p* <.001 (uncorr.) and cluster level of *p* <.05 (FDR corr.). A – Neuronal activation pattern in HC in the 3‐back > 2‐back contrast. B – Neuronal activation pattern in SZ patients in the 3‐back > 2‐back contrast. C – Between‐group analysis in the 3‐back > 2‐back contrast (patients 3‐back > 2‐back versus. controls 3‐back > 2‐back). D – *Brainstem analysis:* Between‐group analysis in the 2‐back versus. 3‐back contrast which was performed at a voxel‐level of *p* <.005 (uncorr.), applying the SVC method using the LC‐mask based on Keren et al., 2009; unmasked brainstem data are depicted. *Abbreviations:* MCC – mid‐cingulate cortex, PCC – posterior cingulate cortex, DLPFC – dorsolateral prefrontal cortex, VLPFC – ventrolateral prefrontal cortex, (pre‐)SMA – (pre) supplementary motor area, AnG – angular gyrus, Caud –caudate nucleus, Cereb – cerebellum, LC – locus coeruleus, MR ‐ nucleus raphe magnus; R – right hemisphere, L – left hemisphere

Overall, we found significant BOLD activation increases from the 2‐ to the 3‐back condition in main regions of the ECN, that is, the DLPFC and VLPFC, in both healthy controls and patients (Figure 3A&B, Table 3A&B). The finding in HC accords well with the common view of the literature. However, we did not expect to find this activation pattern in patients. We also identified an increase in BOLD activation in the PCC, as a main hub of the DMN, from the 2‐ to the 3‐back condition in patients only.

**TABLE 3:** Whole‐brain within‐ and between‐group fMRI analysis in healthy controls and schizophrenia patients in the 3‐back > 2‐back contrast

Region of activation	Right/Left	Brodmann Area	Cluster size	MNI coordinates			*T* value
				X	Y	z	
A: HC 3‐back > 2‐back							
Superior Parietal Cortex	R	7	1,506	45	−62.5	48.5	5.13
Angular Gyrus	R	39		51	−53.5	48.5	4.51
Inferior Parietal Cortex	R	40		54	−40	53	3.99
Medial Frontal Cortex	R	8	1,206	6	29	47	5.08
(pre) Supplementary motor area	R	6		19.5	23	62	4.44
Superior Frontal Cortex	R	8		18	45.5	48.5	4.01
Middle Frontal Cortex	R	10	422	45	48.5	−5.5	4.65
Inferior Frontal Cortex	R	10		42	51.5	3.5	3.79
Dorsolateral Prefrontal Cortex	R	9	378	40.5	17	39.5	3.83
Ventrolateral Prefrontal Cortex	R	45		54	24.5	23	3.64
Dorsolateral Prefrontal Cortex	R	46		52.5	21.5	32	3.61
B: SZ 3‐back > 2‐back							
Middle Temporal Cortex	L	21	86,247	−64.5	−34	−5.5	6.10
Anterior Insula	R	13		48	−44.5	18.5	5.55
Dorsolateral Prefrontal Cortex	R	9		46.5	20	33.5	4.05
Ventrolateral Prefrontal Cortex	R	47		42	26	−7	4.13
Anterior Insula	L	13		−37.5	12.5	−11.5	4.74
Posterior Cingulate Cortex	L	23		−7.7	−43	38	4.25
Putamen	L			−18	14	0.5	5.49
Caudate Nucleus	L			−13.5	0.5	15.5	5.03
Medial Frontal Gyrus	L	8	2,919	−7.5	23	54.5	5.25
(pre) Supplementary motor area	L	6		−13.5	8	54.5	4.17
Superior Frontal Gyrus	R	8		4.5	33.5	53	4.16
C: SZ 3‐back > 2‐back > HC 3‐back > 2‐back							
Superior Temporal Cortex	R	39	65,622	49.5	−44.5	18.5	5.64
Primary Motor Cortex	R	4		16.5	−32.5	59	5.56
(Pre) Supplementary Motor Area	L	6		−9	20	53	4.77
Ventrolateral Prefrontal Cortex	L	44/45		−49.5	15.5	3.5	3.92
Posterior Cingulate Cortex	R	23		1.5	−46	36.5	3.61
Middle cingulate Cortex	L	24		−4.5	5	38	3.68
Caudate Nucleus	L			−15	8	14	3.49
Cerebellum	L		558	−13.5	−40	−20.5	4.79
Somatosensory Cortex	L	2	709	−25.5	−35.5	71	4.22
Angular Gyrus	L	40		−24	−41.5	57.5	4.06
Somatosensory Cortex	L	3		−30	−32.5	59	4.04

Note

Maxima of regions showing significant BOLD activation differences when comparing healthy controls as well as schizophrenia patients in the in the 2‐back versus. 3‐back condition at the whole‐brain level (voxel‐level *p* <.001 uncorr., cluster‐level, *p* <.05, FDR corr.).

Abbreviations: HC – healthy controls, SZ – schizophrenia; posterior cingulate cortex – PCC, Angular Gyrus – AnG, dorsolateral prefrontal cortex – DLPFC, ventrolateral prefrontal cortex – VLPFC, anterior insula – aIN, (pre) supplementary motor area – (pre) SMA, temporal cortex – TCx, cerebellum – Cereb.

Further, we identified BOLD activation increases in SZ patients compared to HC in core nodes of the ECN, for example, in the VLPFC, and the DMN, for example, in the PCC, when analyzing changes from the 2‐ to the 3‐back condition (Figure 3c; Table 3C). The finding of increased PCC activation met our expectation of less deactivation in core regions of the DMN in patients compared to HC with increasing cognitive load.

In addition, we identified an increased BOLD activation pattern in the left LC (x=−4, y=−39, z=−24, t = 3.08, cluster size = 4, *p* <.001 voxel‐level uncorr., *p* <.05 FWE voxel‐level corr.) in SZ patients compared to HC in the 3‐back > 2‐back contrast (Figure 3d). In regard to this analysis, we used the spatially unbiased atlas template (SUIT) preprocessed brainstem/cerebellum functional images, and the LC as anatomical mask image (small volume correction, SVC; mask image based on Keren et al., 2009).

### 3.4 Correlational analyses

**FIGURE 4:**
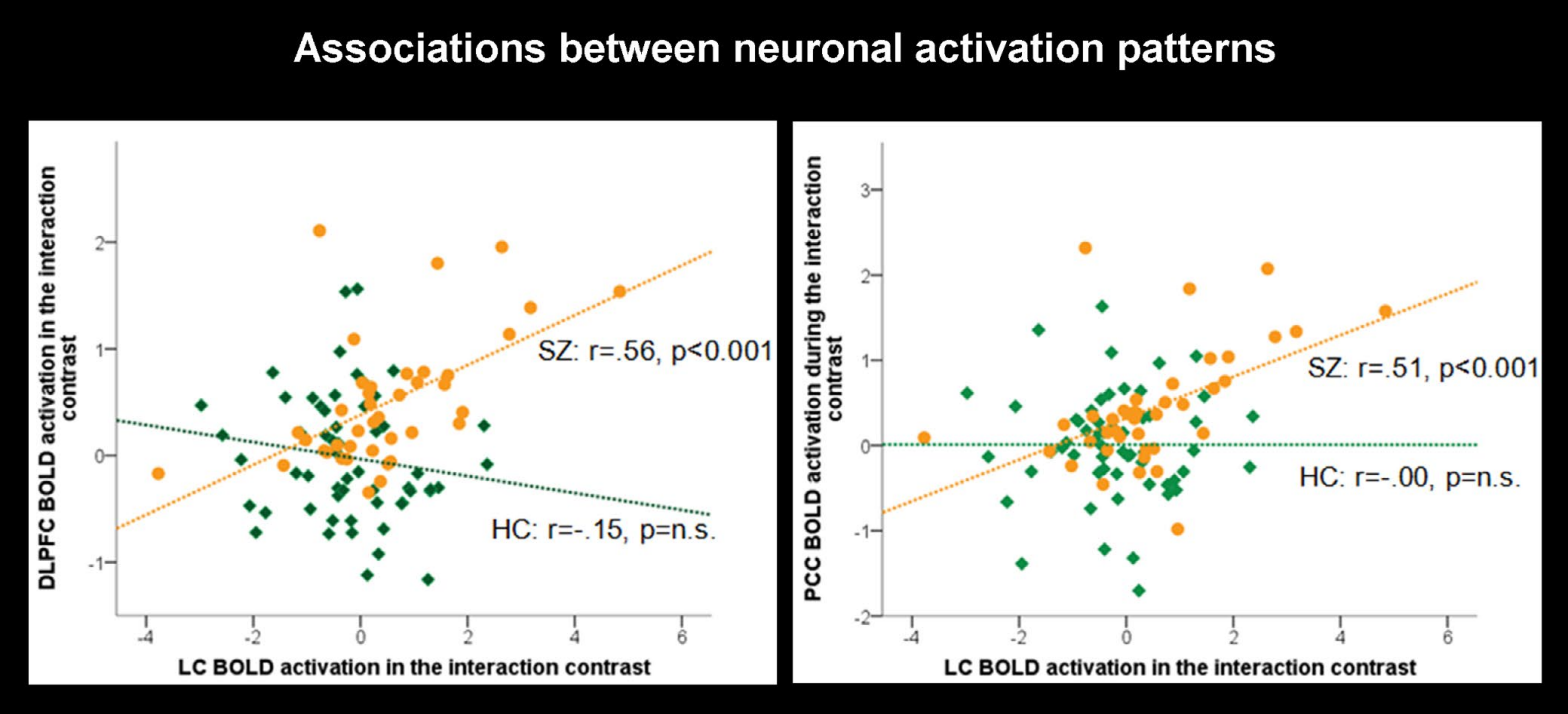
*Associations between neuronal activation patterns*: Correlational analyses between parameter estimates from the DLPFC (x = 40.5, y = 32, z = 35)/PCC (x=−9, y=−50.5, z = 30.5) and LC (x=−4, y=−39, z=−24) in the interaction contrast (SZ 3‐back > 2‐back > HC 3‐back > 2‐back). *Abbreviations:* SZ – schizophrenia (orange color), HC – healthy controls (green color), DLPFC – dorsolateral prefrontal cortex, PCC – posterior cingulate cortex, LC – locus coeruleus, r – Pearson correlation coefficient, n.s. – nonsignificant

To get further insight in the role of the LC regarding dynamic network modulation, we extracted parameter estimates from the DLPFC (x = 40.5, y = 32, z = 35), as a core hub of the ECN, from the PCC (x=−9, y=−50.5, z = 30.5), as a core hub of the DMN, and from the LC (x=−4, y=−39, z=−24) in the between‐group comparison (SZ 3‐back > 2‐back) > (HC 3‐back > 2‐back). We then correlated the LC BOLD activation with the BOLD activation in the DLPFC as well as with the PCC. Most interestingly, in SZ patients, we found significant positive correlations between both DLPFC (r = 0.56, *p* <.001) and PCC (r = 0.51, *p* <.001) BOLD activations with LC BOLD activation which was not the case in the HC group (Figure 4). These findings confirm our supposition of a relation between LC and DLPFC/PCC BOLD activation which varies between patients and controls.

### 3.5 Subgroup analyses

**FIGURE 5:**
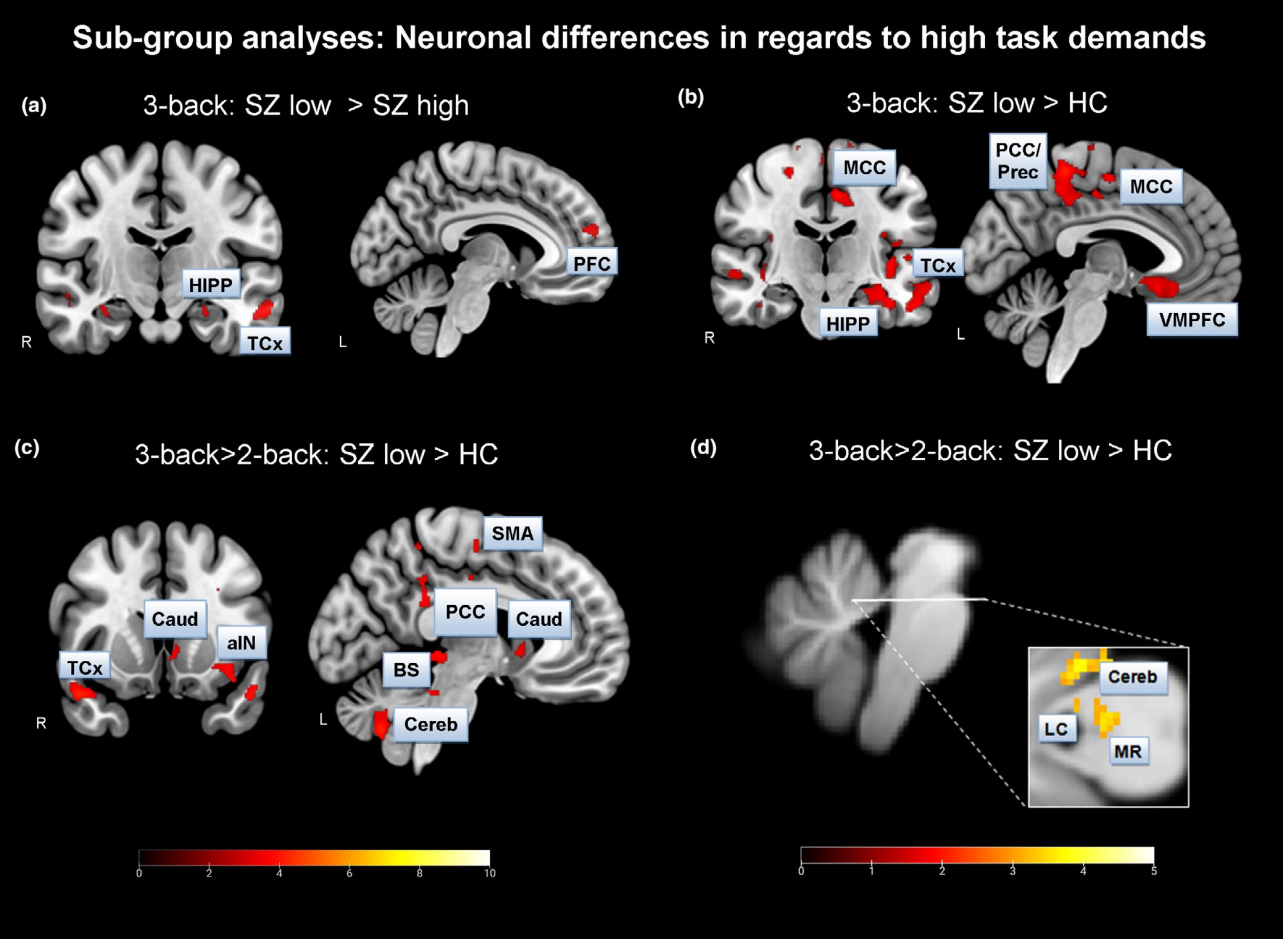
*Subgroup analyses*: A ‐ Patient subgroup analysis in the 3‐back condition was performed on a voxel level of *p* <.005 (uncorr.) and cluster level of *p* <.05 (FDR corr.). B – Low‐performing patients were compared to healthy controls in the hardest task condition. Data were analyzed using a voxel level of *p* <.005 (uncorr.) and a cluster level of *p* <.05 (FDR corr.). C – Low‐performing patients were compared to healthy controls in the 2‐back versus. 3‐back contrast. Data were analyzed using a voxel‐level of *p* <.001 (uncorr.) and a cluster level of *p* <.05 (FDR corr.). D ‐ *Brainstem analysis*: Subgroup analysis was performed on a voxel level of *p* <.005 (uncorr.), applying the SVC method using the LC‐mask based on Keren et al., 2009; unmasked brainstem data are depicted. *Abbreviations:* SZ – schizophrenia, HC – healthy controls, HIPP – hippocampus, Caud – Caudate nucleus, TCx – temporal cortex, MCC – mid‐cingulate cortex, PCC – posterior cingulate cortex, Prec – precuneus, VMPFC – ventromedial prefrontal cortex, aIN – anterior insula, SMA – supplementary motor area, Cereb – cerebellum, LC – locus coeruleus, MR ‐ nucleus raphe magnus; R – right hemisphere, L – left hemisphere

An additional aim of our study was to get insight in performance as a critical differentiating factor. Therefore, we compared the low‐ and high‐performing SZ groups in the demanding 3‐back condition. We identified significant BOLD activation patterns in the temporal cortex (TCx), hippocampus (HIPP), and amygdala as well as a medial prefrontal region (Figure 5a, Table S5A). Further, we found increased BOLD activation, for example, in the PCC/precuneus and VMPFC (Figure 5b, Table S5B), in the low‐performing patient sample compared to HC. Comparing BOLD activation changes from the 2‐ to the 3‐back condition between the low‐performing SZ and the HC group, we found increased BOLD activation, for instance, in the left DLPFC, the anterior/posterior insula (a/pIN), HIPP, caudate nucleus, putamen, amygdala, SMA, PCC/Prec, and TCx (Figure 5c, Table S5C). No significant neuronal activation differences were found by comparing high‐performing patients with control subjects in the most demanding task condition. Thus, the neuronal activation differences between patients and healthy controls seem to be driven by low‐performing patients.

Using the SUIT preprocessed brainstem/cerebellum functional images and the LC as anatomical mask images (SVC; Keren et al., 2009), a significantly increased BOLD activation was detected in the 2‐ versus. 3‐back condition in the low‐performing SZ group compared to the HC group in the left LC (x=−4, y=−39, z=−23, t = 2.82, cluster size = 4, *p* <.005 voxel level (uncorr.), *p* <.05 FWE voxel‐level corr.; Figure 5d).

### 3.6 Subgroup‐specific correlational analyses

We also extracted parameter estimates from the left LC in the between‐group comparison (SZ 3‐back > 2‐back > HC 3‐back > 2‐back) and correlated its BOLD activation with the BOLD signal of both the DLPFC (x = 40.5, y = 32, z = 35) and the PCC (x=−9, y=−50.5, z = 30.5) in the same contrast. Remarkably, in the low‐performing SZ group, we recognized the before found significant positive correlations between both the DLPFC (r = 0.78, *p* <.001) and the PCC (r = 0.76, *p* <.001) with the LC. These relations remained highly significant even after controlling for inferential logic (LC‐DLPFC: r = 0.78, *p* <.001; LC‐PCC: r = 0.75, *p* <.001). However, none of these relations were found in the high‐performing patient group.

## 4 DISCUSSION

The main findings of our present study were increased BOLD activations in the ECN with increasing task difficulty in both SZ and HC. In controls, increasing cognitive demand also led to decreased BOLD activation in the DMN. However, patients seemed not to be capable to maintain this DMN deactivation over conditions. Interestingly, in the interaction contrast between groups and conditions, we have identified increased BOLD activation in the LC in patients compared to HC. LC BOLD activation significantly correlated with both, the main hub of the ECN, that is, the DLPFC, as well as of the DMN, that is, the PCC. In a subgroup analysis, we discovered that most of the SZ versus. HC group differences were driven by the low‐performing patient group. Most remarkably, in the low‐performing patient group we found the LC being significantly correlated with both the DLPFC and the PCC once again. Thus, the LC seems to play an exquisite role in modulating neuronal activity of the ECN and DMN in a 3‐back working memory task and might contribute to cognitive impairments in SZ.

Overall, we confirmed our hypothesis with respect to the behavioral results. Patients performed increasingly worse with rising task demands. Nevertheless, it was surprising that patients performed also quite well on both the 2‐ and 3‐back conditions. Further, as indicated by scores on symptom rating scales, patients were not in an acute state of illness which might also contribute to the good performance.

Our finding of increased ECN BOLD activation, especially in the DLPFC, with increases in working memory load in healthy controls goes along with former research findings (Jansma et al., 2004). In patients with schizophrenia, we also observed increased BOLD activations in core regions of the ECN in regard of the 3‐ versus. 2‐back contrast which was rather surprising. Various research groups are discussing whether PFC hypo‐ or hyper‐activation observed in schizophrenia patients is related to the poor performance on cognitive tasks which is, in general, a feature of this patient group (Jansma et al., 2004; Jiang et al., 2015). Weinberger et al., (2001) proposed that even when patients with schizophrenia are able to keep up with processing demands, they do so less efficiently than controls and need to work harder to keep up which requires the recruitment of greater and/or less focused neuronal activity. Callicott et al., (2003) and Manoach (2003) have further proposed that there is an inverted U‐shaped function between working memory load and PFC activation, such that increasing task demands are first associated with increasing activation, which then falls off after the subject's working memory capacity is exceeded. They argue that this curve is shifted to the left in schizophrenia, causing patients first to show more activation than controls at low task demands, but then to reach their maximum capacity earlier and thereafter show less activation. Another possibility is that increased PFC activation in schizophrenia reflects a failure of DMN deactivation (Greicius et al., 2003; Gusnard et al., 2001; Raichle et al., 2001).

In our present study, we consistently found core hubs of the DMN, that is, the PCC, being deactivated in both healthy controls and schizophrenia patients. However, in patients we found BOLD activation increases in the 3‐ versus. 2‐back contrast in core hubs of the DMN. Thus, with increasing task demands patients seemed not to be capable to maintain DMN deactivation over conditions.

The ECN is needed to maintain task goals and prevent lapses of attention by suppressing the DMN (Unsworth and Robinsn, 2017). Accordingly, former research has suggested that individuals with higher working memory capacities demonstrate a stronger anti‐correlation between ECN and DMN (Keller et al., 2015). Besides, Kelly et al., (2008) and Esterman et al., (2013) have found that greater DMN BOLD activation is associated with greater variability in response times. This finding suggests an association between attentional lapses and DMN activation. In fact, Kelly et al. found that the greater the negative correlation between ECN and DMN, the more consistent behavior was. The weaker the negative correlation between ECN and DMN, the more inconsistent behavior became.

However, when tasks require retrieval or access of information from memory, the ECN and DMN were also found to act together (Konishi et al., 2015; Smallwood et al., 2013; Spreng et al., 2014; Vatansever et al., 2015). Konishi et al., (2015) used a spatial n‐back task and found, among others, increased BOLD activations in DMN regions. In this context, DMN activation was proposed to be crucial for, for example, task judgments depending on recollections based on memory. Spreng et al., (2014) also demonstrated that the DMN and ECN cooperate to perform a working memory task.

Considering our results, in schizophrenia patients we found both increases in ECN BOLD activation accompanying increasing task demands and DMN BOLD deactivation. However, patients seemed not to be capable to maintain this DMN deactivation with increasing memory load. Further, we found a strong positive correlation between the DLPFC and PCC which are core hubs of the ECN/DMN, respectively. Most interestingly, we have also found increased BOLD activation in the locus coeruleus in SZ patients compared to HC.

The LC‐NE system plays a pivotal role in cognitive control (Unsworth & Robison, 2017). It is also known that working memory is highly dependent upon noradrenergic neurotransmission in the PFC. For instance, delay‐related firing, an electrophysiological correlate of working memory, occurs in prefrontal neurons in response to a behaviorally relevant stimulus. This type of activation of PFC neurons is potentiated by activation of the *α*2A receptor and diminished by its antagonists. Thus, working memory might be improved or impaired (Wang et al., 2007). Further, the LC was found to be functionally integrated into the ECN in the resting condition (Bär et al., 2016). Regarding our present results, we suppose that the LC directly modulates proper ECN function by indirectly impacting on the DMN during task performance. Thus, an intact LC‐NE system supports working memory function by influencing large‐scale brain networks. One might even suggest that a highly synchronous neuronal activation between both the LC‐NE system and core hubs of the ECN supports cognitive functioning. The network reset theory of Bouret and Sara (2005) suggests that a major function of NE is to reset ongoing brain activity in order to synchronize large‐scale brain networks in preparation for responding. This, together with theories that the P3, which is an event‐related potential being influenced by conflict monitoring and adaptation, is the result of NE release in the cortex suggests that network reset is a potential mechanism leading to coordinated cortical responses following task‐relevant stimuli (Rawls et al., 2020). Subsequently, decreases in task performance, that is, with increasing cognitive demand, might also be driven by a desynchronization of the LC‐NE system and the ECN. In consequence, attentional lapses are probable as well as increases in DMN BOLD activation and a decrease in ECN BOLD activity. What is more, it is assumed that individuals with high attentional control abilities are also characterized by enhanced cognitive control capacities, for example, working memory capacities, than individuals with low attentional control abilities. Thus, during cognitive task performance, desynchronization phenomena might sooner be present in individuals with lower cognitive capacities and reduced attentional control (Unsworth & Robison, 2017). This might be the case in individuals suffering from schizophrenia. As a result, on the neuronal level one might still see increased LC and ECN BOLD activation by, however, simultaneously increasing DMN BOLD activation. In this case, depending on task challenges, behavioral performance will also very likely worsen.

However, when task demands are either too easy or too challenging performance worsens (Aston‐Jones & Cohen, 2005; Berridge & Waterhouse, 2003; Chamberlain & Robbins, 2013; Ramos & Arnsten, 2007). Specifically, when tonic LC activity is low (hypo‐arousal), individuals are inattentive, nonalert, and disengaged from the current task leading to poor behavioral performance and little to none phasic LC activity in response to task‐relevant stimuli. As tonic LC activity increases to an intermediate range (phasic response), attention becomes more focused and behavioral performance improves. However, as tonic LC activity increases further, individuals experience a more distractible attentional state (hyper‐arousal and stress) leading to task disengagement, lowered LC phasic activity, and a reduction in behavioral performance. Animal experiments and psychopharmacological studies provide evidence in support of the noted inverted U relationship between the LC‐NE system and behavioral performance (Aston‐Jones & Cohen, 2005; Berridge & Waterhouse, 2003; Chamberlain & Robbins, 2013; Ramos & Arnsten, 2007). However, it might be assumed that hypo‐ and hyper‐arousal states of the LC are accompanied by reduced large‐scale brain network synchrony.

Subgroup analyses of our present study were performed to get further insight into neuronal activation changes, how these are related to LC BOLD activation, and whether performance is a critical differentiating factor which should be accounted for. Indeed, the subgroup analyses were very intriguing. We found the low‐performing SZ group accounting for most of the between‐group differences even though patient groups did not differ in pathology and medication equivalent doses. The high‐performing SZ group exhibited similar neuronal activation patterns such as healthy controls. Even correlational analyses were driven by the low‐performing patient group and, as was the case in the HC group, nonsignificant in the high‐performing SZ group. Even after controlling for inferential logic, results remained constant.

Contrary to our expectation that LC BOLD activation is positively related to DLPFC and negatively related to PCC BOLD activation in HC but not in patients, we found significant relations between this brainstem structure and the core hub of the ECN as well as DMN in SZ patients. Further, no significant correlations were found in healthy controls. These relations in SZ patients, in particular the low‐performing ones, can be ascribed as a constant effort of the LC‐NE system to sustain or enhance ECN BOLD activation while deactivating DMN activity as a disruptive neuronal network activation. This relation is interpreted in order to optimize the attentional focus to improve or maintain task performance. In contrast, in high‐performing individuals this continuous LC firing is not necessary because an optimal neuronal network interaction might have been initially initiated and maintained over the task. Finally, another explanation might be that LC allocates additional resources when ECN activation alone fails to optimally perform on a task. Thus, low‐performing patients might strongly try to retrieve information from their memory to recall seen letters. In this case, ECN and DMN co‐activation with the LC being the driving structure might be a helpful strategy.

Some study limitations should be discussed. Although sample sizes seem to be sufficient regarding the overall group analyses, formed subgroups were small, and thus, those results lack power and should be interpreted with some caution. Furthermore, future studies should pay attention to a more balanced sample distribution, especially regarding the ratio between female and male subjects. Moreover, it would be of immense interest to study healthy controls in a low‐performance condition and see whether found neuronal patterns are similar to the ones found in patients. This is necessary to get further insight into the exact role of the LC‐NE system regarding neuronal and large‐scale brain network modulation. Unfortunately, in the present study we were not able to form a low‐performing HC group because just four healthy controls could have assigned to this group. In this context, we want to note another disadvantage of the study. Because of the high‐performance scores, we cannot rule out that task demands were not challenging enough to elucidate the particular role of the LC‐NE system in light of large‐scale brain network modulation. However, task demands allowed the allocation of balanced patient subgroups which lead to very interesting results which should be kept in mind. Furthermore, to analyze dynamic changes in neuronal activation over the entire length of the task as well as condition‐specific, an event‐related design would be most helpful. Thus, the dynamic role of the LC‐NE system and its relation to large‐scale brain networks could be determined further. Another limitation is the analysis of merely medicated patients with schizophrenia. Although we described patient subgroups in terms of drug treatment and found no significant differences in their distribution, an influence of medication on neuronal networks and corresponding neurotransmitter systems cannot be excluded. Accordingly, future studies should examine sufficiently large subgroups with respect to drug therapy to better understand the influence of psychopharmacological treatment on neuronal networks and neurotransmitter systems. Nevertheless, in our present study we divided the sample in high‐ and low‐performing patients and found differences even though patient subgroups did not differ in terms of CPZ equivalent doses, drug distribution, or psychopathology.

In conclusion, our study adds to the growing body of research that demonstrates that higher‐order tasks cannot be attributed to a single neural network. Instead, proper cognitive function depends on the coordinated activity of multiple brain networks in a flexible fashion, most probable being driven by the LC‐NE system.

However, one should keep in mind that we have only considered one memory task and that we observed correlations which do not allow causal conclusions. Nonetheless, present results indicate interesting patterns of network interactions and, thus, can be considered as a basis for subsequent studies allowing deeper insights using approaches such as network‐based analyses, Granger Causality, or Dynamic Causal Modeling.

### PEER REVIEW

The peer review history for this article is available at https://publons.com/publon/10.1002/brb3.2130.

## Supporting information

Supplementary Material

## Data Availability

The data that support the findings of this study are available from the corresponding author upon reasonable request.
