# Supplementary material for: Working memory in schizophrenia: The role of the locus coeruleus and its relation to functional brain networks
Source: Brain Behav. 2021 Mar 30;11(5):e02130. doi: 10.1002/brb3.2130 (PMC8119871; doi:10.1002/brb3.2130)
Supplement: Supplementary file 1 — Supplementary Material [file BRB3-11-e02130-s001.docx]

**SUPPLEMENT**

*Table S1. Antipsychotic medication (second-generation antipsychotics)*

| Antipsychotic medication | Number of patients |
| --- | --- |
| *All patients* |  |
| Olanzapin | 21 |
| Aripiprazol | 4 |
| Risperidon | 11 |
| Quetiapin | 3 |
| Amisulprid | 4 |
| *Low-performing patients* |  |
| Olanzapin | 11 |
| Aripiprazol | 1 |
| Risperidon | 8 |
| Quetiapin | 1 |
| Amisulprid | 2 |
| *High-performing patients* |  |
| Olanzapin | 10 |
| Aripiprazol | 3 |
| Risperidon | 3 |
| Quetiapin | 2 |
| Amisulprid | 2 |

*Table S2: Whole-brain within-group fMRI analysis in healthy controls in the 1-back vs. x-back, 2-back vs. x-back and 3-back vs. x-back condition*

| Region of activation | Right/  Left | Brodmann Area | Cluster size | MNI coordinates | | | *T* value |
| --- | --- | --- | --- | --- | --- | --- | --- |
|  |  |  |  | x | y | z |  |
| Healthy controls 1-back > x-back | | | | | | | |
| Inferior Parietal Cortex | L | 40 | 1974 | -31.5 | -46 | 44 | 5.00 |
| Inferior Parietal Lobule | L | 40 |  | -40.5 | -44.5 | 39.5 | 4.37 |
| Superior Parietal Lobule | L | 7 |  | -31.5 | -59.5 | 47 | 4.36 |
| Inferior Parietal Lobule | R | 40 | 2254 | 40.5 | -46 | 50 | 4.90 |
| Angular Gyrus | R | 39 |  | 33 | -52 | 45.5 | 4.79 |
| Healthy controls 2-back > x-back | | | | | | | |
| Inferior Parietal Cortex | L | 40 | 25191 | -31.5 | -46 | 41 | 14.91 |
| Inferior Parietal Cortex | R | 40 |  | 40.5 | -40 | 44 | 14.77 |
| Superior Parietal Cortex | R | 7 |  | 15 | -68.5 | 57.5 | 14.56 |
| (pre-) Supplementary Motor Area | L | 6 | 59400 | -25.5 | 3.5 | 56 | 13.33 |
| Dorsolateral Prefrontal Cortex | R | 9 |  | 39 | 30.5 | 36.5 | 11.42 |
| Cerebellum | R |  | 26953 | 36 | -67 | -25 | 11.12 |
| Cerebellum | L |  |  | -27 | -62.5 | -29.5 | 10.97 |
| Middle Temporal Gyrus | R | 37 | 1262 | 57 | -53.5 | -11.5 | 7.76 |
| Middle Temporal Gyrus | R | 21 |  | 60 | -37 | -11.5 | 6.32 |
| Healthy controls 2-back < x-back | | | | | | | |
| Posterior Cingulate Cortex | L | 23 | 678 | -3 | -52 | 20 | 10.79 |
| Precuneus | L | 31 | 2215 | -9 | -49 | 32 | 9.60 |
| Cerebellum | R |  | 9524 | 28.5 | -86.5 | -34 | 8.99 |
| Middle Temporal Cortex | L | 39 |  | -43.5 | -61 | 29 | 8.57 |
| Subgenual Anterior Cingulate Cortex | L | 25 | 5681 | -1.5 | 6.5 | -10 | 8.54 |
| Medial Frontal Cortex | M | 10 |  | 0 | 59 | 15.5 | 8.40 |
| Ventromedial Prefrontal Cortex | L | 10/11 |  | -3 | 15.5 | -11.5 | 8.26 |
| Middle Temporal Cortex | R | 21 | 2507 | 60 | 3.5 | -22 | 7.49 |
| Fusiform Gyrus | R | 19 |  | 28.5 | -56.5 | -5.5 | 6.41 |
| Middle Temporal Cortex | L | 21 | 1533 | -64.5 | -11.5 | -13 | 7.32 |
| Ventrolateral Prefrontal Cortex | L | 47 |  | -43.5 | 24.5 | -14.5 | 6.43 |
| Fusiform Gyrus | L | 20 |  | -60 | -14.5 | -25 | 6.35 |
| Parahippocampal Gyrus | L | 36 | 2467 | -27 | -41.5 | -10 | 6.85 |
| Hippocampus | L | 28 |  | -24 | -19 | -16 | 5.48 |
| Parahippocampal Gyrus | L | 19 |  | -28.5 | -52 | -7 | 5.43 |
| Inferior Parietal Cortex | R | 40 | 594 | 55.5 | -26.5 | 23 | 6.35 |
| Posterior Insula | R |  |  | 39 | -19 | 0.5 | 4.96 |
| Superior Temporal Cortex | R | 22 |  | 55.5 | 0.5 | 5 | 4.63 |
| Cuneus | R | 19 | 282 | 15 | -91 | 30.5 | 5.34 |
| Cuneus | R | 19 |  | 25.5 | -88 | 30.5 | 4.85 |
| Middle Occipital Cortex | R | 18 |  | 27 | -92.5 | 18.5 | 4.60 |
| Superior Temporal Cortex | L | 38 | 928 | -33 | 6.5 | -19 | 5.26 |
| Amygdala | L |  |  | -24 | -1 | -17.5 | 4.15 |
| Superior Temporal Cortex | L | 38 |  | -39 | 0.5 | -13 | 3.51 |
| Precentral Gyrus | L | 4 | 422 | -36 | -23.5 | 63.5 | 5.14 |
| Precentral Gyrus | L | 4 | 276 | -34.5 | -23.5 | 51.5 | 4.47 |
| Postcentral Gyrus | L | 5 |  | -22.5 | -44.5 | 68 | 5.00 |
| Cuneus | L | 19 | 238 | -15 | -94 | 27.5 | 5.00 |
| Cuneus | L | 19 | 680 | -7.5 | -91 | 30.5 | 4.06 |
| Middle Occipital Cortex | L | 18 |  | -21 | -95.5 | 20 | 3.30 |
| Angular Gyrus | R | 39 |  | 54 | -64 | 30.5 | 4.98 |
| Inferior Parietal Cortex | L | 40 | 272 | -54 | -29.5 | 23 | 4.62 |
| Insula | L | 13 |  | -48 | -22 | 20 | 4.46 |
| Healthy controls 3-back > x-back | | | | | | | |
| Inferior Parietal Cortex | R | 40 | 23671 | 43.5 | -40 | 47 | 16.15 |
| Inferior Parietal Cortex | L | 40 |  | -39 | -50.5 | 48.5 | 15.65 |
| Medial Frontal Cortex | R | 8 | 62930 | 4.5 | 23 | 45.5 | 13.70 |
| (pre-) Supplementary Motor Area | L | 6 |  | -27 | 5 | 57.5 | 13.47 |
| (pre-) Supplementary Motor Area | R | 6 |  | 33 | 6.5 | 62 | 13.29 |
| Cerebellum | R |  | 24277 | 36 | -65.5 | -25 | 12.50 |
| Cerebellum | L |  |  | -36 | -65.5 | -28 | 12.24 |
| Inferior Temporal Cortex | R | 20 | 1776 | 60 | -47.5 | -11.5 | 9.14 |
| Middle Temporal Cortex | R | 21 |  | 60 | -37 | -11.5 | 8.99 |
| Visual Cortex | R | 17 | 293 | 18 | -98.5 | -2.5 | 4.66 |
| Visual Gyrus | R | 18 | 23671 | 19.5 | -88 | 0.5 | 4.04 |
| Healthy controls 3-back < x-back | | | | | | | |
| Posterior Cingulate Cortex | L | 23 | 21868 | -4.5 | -53.5 | 20 | 10.95 |
| Precuneus | L | 31 |  | -9 | -50.5 | 30.5 | 9.66 |
| Paracentral Cortex | L | 5 |  | -10.5 | -32.5 | 47 | 7.95 |
| Ventromedial Prefrontal Cortex | L | 10/11 | 7991 | -1.5 | 8 | -11.5 | 8.56 |
| Medial Frontal Cortex | L | 10 |  | -1.5 | 59 | 14 | 8.43 |
| Superior Frontal Cortex | L | 8 |  | -12 | 41 | 54.5 | 7.72 |
| Cerebellum | R |  | 650 | 28.5 | -86.5 | -34 | 8.52 |
| Middle Temporal Cortex | L | 39 | 2905 | -43.5 | -61 | 29 | 8.52 |
| Postcentral Gyrus | R | 40 | 12940 | 55.5 | -22 | 17 | 8.26 |
| Superior Temporal Cortex | R | 41 |  | 49.5 | -29.5 | 17 | 7.84 |
| Posterior Insula | R |  |  | 39 | -19 | 0.5 | 7.56 |
| Parahippocampal Cortex | L | 36 | 7225 | -24 | -43 | -10 | 8.23 |
| Posterior Insula | L | 13 |  | -39 | -20.5 | 2 | 7.38 |
| Parahippocampal Cortex | L | 19 |  | -30 | -50.5 | -7 | 7.30 |
| Middle Temporal Cortex | L | 21 | 2308 | -63 | -8.5 | -13 | 7.78 |
| Ventrolateral Prefrontal Cortex | L | 47 | 337 | -43.5 | 27.5 | -13 | 6.38 |
| Cuneus | L | 19 | 677 | -15 | -94 | 27.5 | 6.36 |
| Cuneus | R | 19 | 1950 | 10.5 | -88 | 35 | 5.89 |
| Middle Temporal Cortex | R | 39 |  | 57 | -61 | 8 | 5.46 |

*Maxima of regions showing significant BOLD activation differences when comparing the three conditions with the control condition at the whole‐brain level in healthy control subjects (voxel‐level p<0.001 uncorr., cluster‐level, p<0.05, FDR corr.); Abbreviations used in the text: posterior cingulate cortex – PCC, precuneus – prec, anterior cingulate cortex – ACC, dorsolateral prefrontal cortex – DLPFC, ventrolateral prefrontal cortex – VLPFC, ventromedial prefrontal cortex – VMPFC, anterior insula – aIN, (pre-)supplementary motor area – (pre-)SMA, temporal cortex – TCx, cerebellum – Cereb, Thal – thalamus, hippocampus – HIPP, caudate nucleus - Caud.*

*Table S3: Whole-brain within-group fMRI analysis in patients with schizophrenia in the 1-back vs. x-back, 2-back vs. x-back and 3-back vs. x-back condition*

| Region of activation | Right/  Left | Brodmann Area | Cluster size | MNI coordinates | | | *T* value |
| --- | --- | --- | --- | --- | --- | --- | --- |
|  |  |  |  | x | y | z |  |
| Patients 1-back > x-back | | | | | | | |
| Inferior Parietal Cortex | R | 40 | 4226 | 40.5 | -52 | 59 | 5.73 |
| Precuneus | R | 7 |  | 30 | -50.5 | 47 | 5.55 |
| Superior Parietal Cortex | L | 7 | 3560 | -28.5 | -56.5 | 48.5 | 5.12 |
| Inferior Parietal Cortex | L | 40 |  | -42 | -52 | 53 | 5.08 |
| Superior Parietal Cortex | L | 7 |  | -28.5 | -58 | 57.5 | 5.03 |
| Superior Frontal Cortex | R | 8 | 403 | 3 | 17 | 50 | 4.58 |
| Cerebellum | L |  | 423 | -40.5 | -68.5 | -29.5 | 4.45 |
| Dorsolateral Prefrontal Cortex | R | 9 | 1694 | 48 | 24.5 | 30.5 | 4.42 |
| Middle Frontal Cortex | R | 8 |  | 52.5 | 9.5 | 41 | 4.37 |
| Dorsolateral Prefrontal Cortex | R | 9 |  | 40.5 | 32 | 32 | 4.24 |
| (Pre-) Supplementary Motor Area | L | 6 | 481 | -40.5 | 3.5 | 33.5 | 4.29 |
| Middle Frontal Cortex | L | 8 |  | -51 | 12.5 | 39.5 | 3.44 |
| Cerebellum | L |  | 592 | -4.5 | -80.5 | -19 | 4.28 |
| Fusiform Gyrus | L | 37 | 794 | -48 | -59.5 | -10 | 4.26 |
| Cerebellum | R |  | 293 | 45 | -64 | -17.5 | 3.98 |
| Fusiform Gyrus | R | 37 |  | 55.5 | -53.5 | -16 | 3.70 |
| Inferior Temporal Gyrus | R | 20 |  | 60 | -37 | -16 | 3.58 |
| Patients 2-back > x-back | | | | | | | |
| Inferior Parietal Cortex | R | 40 | 12789 | 40.5 | -43 | 48.5 | 8.45 |
| Superior Parietal Cortex | L | 7 |  | -24 | -64 | 47 | 8.17 |
| Superior Parietal Cortex | R | 7 |  | 30 | -67 | 50 | 7.74 |
| (Pre-) Supplementary Motor Area | L | 6 | 4357 | -42 | 3.5 | 33.5 | 7.25 |
| Dorsolateral Prefrontal Cortex | L | 9 |  | -45 | 30.5 | 35 | 6.09 |
| (Pre-) Supplementary Motor Area | R | 6 | 2000 | 1.5 | 15.5 | 50 | 7.09 |
| (Pre-) Supplementary Motor Area | L | 6 |  | -3 | 8 | 54.5 | 6.39 |
| (Pre-) Supplementary Motor Area | R | 6 | 6721 | 28.5 | -1 | 60.5 | 6.75 |
| Dorsolateral Prefrontal Cortex | R | 9 |  | 40.5 | 35 | 35 | 6.75 |
| Anterior Insula | L | 13 | 620 | -31.5 | 21.5 | 2 | 4.99 |
| Cerebellum | L |  | 615 | -31.5 | -62.5 | -26.5 | 4.84 |
| Middle Frontal Cortex | L | 10 | 746 | -37.5 | 53 | 17 | 4.65 |
| Dorsolateral Prefrontal Cortex | L | 46 |  | -39 | 45.5 | 8 | 4.05 |
| Anterior Insula | R | 13 | 403 | 31.5 | 23 | 5 | 4.53 |
| Ventrolateral Prefrontal Cortex | R | 47 |  | 37.5 | 21.5 | -2.5 | 3.84 |
| Cerebellum | R |  | 500 | 37.5 | -64 | -23.5 | 4.30 |
| Patients 2-back < x-back | | | | | | | |
| Medial Frontal Cortex | L | 10 | 8989 | -4.5 | 60.5 | 6.5 | 7.03 |
| Ventromedial Prefrontal Cortx | L | 10 |  | -1.5 | 51.5 | -5.5 | 5.77 |
| Subgenual Anterior Cingulate | L | 25 |  | -1.5 | 5 | -10 | 5.74 |
| Middle Temporal Cortex | L | 39 | 2260 | -49.5 | -64 | 24.5 | 6.90 |
| Middle Temporal Cortex | R | 21 | 8647 | 58.5 | -1 | -22 | 6.88 |
| Superior Temporal Cortex | R | 38 |  | 37.5 | 21.5 | -37 | 5.72 |
| Posterior Cingulate Cortex | L | 31 | 8936 | -4.5 | -43 | 35 | 6.82 |
| Precuneus | L | 31 |  | -10.5 | -50.5 | 30.5 | 6.24 |
| Cerebellum | R |  | 856 | 27 | -82 | -31 | 6.71 |
| Middle Temporal Cortex | L | 21 | 3809 | -54 | 6.5 | -28 | 6.54 |
| Ventrolateral Prefrontal Cortex | L | 47 |  | -46.5 | 29 | -11.5 | 5.97 |
| Parahippocampal Gyrus | L | 36 | 1952 | -24 | -37 | -14.5 | 5.68 |
| Superior Temporal Cortex | R | 39 | 911 | 45 | -53.5 | 23 | 5.49 |
| Superior Temporal Cortex | R | 22 |  | 49.5 | -55 | 14 | 3.54 |
| Inferior Parietal Cortex | R | 40 | 861 | 54 | -26.5 | 23 | 5.14 |
| Superior Temporal Cortex | R | 41 |  | 42 | -31 | 14 | 4.46 |
| Postcentral Gyrus | R | 40 |  | 54 | -22 | 15.5 | 3.97 |
| Postcentral Gyrus | L | 5 | 445 | -21 | -43 | 71 | 4.66 |
| Superior Parietal Cortex | L | 5 |  | -21 | -43 | 59 | 3.69 |
| Superior Parietal Cortex | R | 7 | 552 | 16.5 | -43 | 62 | 4.29 |
| Postcentral Gyrus | R | 3 |  | 21 | -35.5 | 63.5 | 4.02 |
| Precentral Gyrus | L | 4 | 647 | -37.5 | -19 | 57.5 | 4.06 |
| Cerebellum | L |  |  | -36 | -20.5 | 2 | 3.71 |
| Superior Temporal Cortex | L | 22 |  | -49.5 | -8.5 | 5 | 3.58 |
| Patients 3-back > x-back | | | | | | | |
| Inferior Parietal Cortex | R | 40 | 47755 | 39 | -44.5 | 47 | 11.70 |
| Superior Parietal Cortex | R | 7 |  | 33 | -59.5 | 44 | 10.99 |
| Precentral Gyrus | L | 6 | 48425 | -42 | 3.5 | 33.5 | 10.42 |
| (Pre-) Supplementary Motor Area | R | 6 |  | 1.5 | 15.5 | 51.5 | 9.90 |
| Dorsolateral Prefrontal Cortex | L | 9 |  | -43.5 | 30.5 | 33.5 | 9.37 |
| Thalamus | L |  | 1545 | -12 | -5.5 | 14 | 5.25 |
| Thalamus | R |  |  | 7.5 | -22 | 11 | 4.85 |
| Cingulate Gyrus | L | 24 | 417 | -3 | 2 | 29 | 4.19 |
| Cingulate Gyrus | R | 23 |  | 7.5 | -11.5 | 32 | 3.75 |
| Patients 3-back < x-back | | | | | | | |
| Ventromedial Prefrontal Cortex | L | 10 | 635 | -4.5 | 60.5 | 6.5 | 5.67 |
| Superior Frontal Cortex | L | 10 |  | -7.5 | 65 | 20 | 3.76 |
| Posterior Cingulate Cortex | L | 31 | 2217 | -3 | -52 | 26 | 5.63 |
| Posterior Cingulate Cortex | R | 31 |  | 6 | -52 | 27.5 | 5.05 |
| Precuneus | L | 31 |  | -12 | -61 | 20 | 4.76 |
| Middle Temporal Cortex | L | 39 | 468 | -42 | -62.5 | 26 | 4.65 |
| Middle Temporal Cortex | L | 39 |  | -45 | -70 | 24.5 | 4.02 |

*Maxima of regions showing significant BOLD activation differences when comparing the three conditions with the control condition at the whole‐brain level in patients with schizophrenia (voxel‐level p<0.001 uncorr., cluster‐level, p<0.05, FDR corr.); Abbreviations used in the text: posterior cingulate cortex – PCC, precuneus – prec, anterior cingulate – ACC, dorsolateral prefrontal cortex – DLPFC, ventrolateral prefrontal cortex – VLPFC, ventromedial prefrontal cortex – VMPFC, anterior insula – aIN, (pre-)supplementary motor area – (pre-)SMA, temporal cortex – TCx, cerebellum – Cereb, Thal – thalamus, hippocampus – HIPP, caudate nucleus - Caud.*

*Table S4: Whole-brain between-group fMRI analyses in the 2-back and 3-back condition*

| Region of activation | Right/  Left | Brodmann Area | Cluster size | MNI coordinates | | | *T* value |
| --- | --- | --- | --- | --- | --- | --- | --- |
|  |  |  |  | x | y | z |  |
| A: HC 2-back > SZ 2-back | | | | | | | |
| Putamen | L |  | 811 | -18 | 12.5 | 2 | 5.25 |
| Caudate Nucleus | M |  |  | 0 | 8 | 5 | 4.36 |
| Thalamus | L |  |  | -1.5 | -2.5 | 6.5 | 4.15 |
| Cerebellum | L |  | 1367 | -15 | -35.5 | -22 | 4.66 |
| Middle Frontal Cortex | L | 6 | 513 | -31.5 | 0.5 | 47 | 4.29 |
| B: SZ 3-back > HC 3-back | | | | | | | |
| Angular Gyrus | L | 40 | 929 | -55.5 | -55 | 38 | 4.55 |
| Inferior Parietal Cortex | L | 40 |  | -61.5 | -40 | 33.5 | 4.46 |
| Superior Temporal Cortex | L | 39 |  | -45 | -53.5 | 29 | 4.08 |
| Precuneus | L | 31 | 658 | -4.5 | -38.5 | 60.5 | 4.54 |
| Posterior Cingulate Cortex | L | 31 |  | -3 | -37 | 42.5 | 4.16 |
| Primary Motor Cortex | R | 4 | 514 | 6 | -29.5 | 75.5 | 4.41 |

*Maxima of regions showing significant BOLD activation differences when comparing healthy controls and schizophrenia patients in the 2-back and 3-back conditions at the whole‐brain level (voxel‐level p<0.001 uncorr., cluster‐level, p<0.05, FDR corr.); Abbreviations used in the text: posterior cingulate cortex – PCC, precuneus – prec, angular gyrus – AnG, temporal cortex – TCx, cerebellum – Cereb, caudate nucleus - Caud.*

*Table S5: Whole-brain between sub-group fMRI analyses in the 2-back and 3-back condition*

| Region of activation | Right/  Left | Brodmann Area | Cluster size | MNI coordinates | | | *T* value |
| --- | --- | --- | --- | --- | --- | --- | --- |
|  |  |  |  | x | y | z |  |
| A: 3-back: SZ low > SZ high | | | | | | | |
| Hippocampus | L |  | 919 | -27 | -22 | -13 | 4.14 |
| Inferior Temporal Cortex | L | 21 | 1682 | -62 | -13 | -18 | 4.12 |
| Middle Temporal Cortex | L | 38 |  | -48 | 5 | -24 | 4.04 |
| Superior Temporal Cortex | L | 38 |  | -45 | 10 | -16 | 3.49 |
| Middle Temporal Cortex | R | 21 | 1602 | 57 | 1 | -30 | 3.92 |
| Hippocampus | R |  |  | 32 | -13 | -19 | 3.53 |
| Middle Temporal Cortex | R | 21 |  | 62 | 1 | -15 | 3.51 |
| Superior Frontal Cortex | R | 9/10 | 1018 | 6 | 59 | 23 | 3.66 |
| Superior Frontal Cortex | L | 9/10 |  | -6 | 55 | 20 | 3.51 |
| B: 3-back: SZ low > HC | | | | | | | |
| Middle Temporal Cortex | L | 21 | 7840 | -60 | -28 | -12 | 4.34 |
| Hippocampus | L |  |  | -29 | -24 | -13 | 4.20 |
| Parahippocampal Cortex | L | 36 |  | -23 | -42 | -9 | 4.17 |
| Supramarginal Cortex | L | 40 | 2422 | -56 | -54 | 29 | 4.30 |
| Superior Temporal Cortex | L | 39 |  | -44 | -52 | 31 | 3.93 |
| Inferior Parietal Lobule | L | 40 |  | -54 | -57 | 38 | 3.79 |
| Paracentral Lobule | L | 5 | 4955 | -5 | -39 | 61 | 4.26 |
| Paracentral Lobule | L | 31 |  | -8 | -21 | 44 | 4.17 |
| Lingual Cortex | R | 19 | 7982 | 29 | -58 | -3 | 4.26 |
| Fusiform Cortex | R | 37 |  | 32 | -49 | -7 | 4.25 |
| Fusiform Cortex | R | 19 |  | 30 | -69 | -6 | 4.22 |
| Medial Frontal Cortex | M | 25 | 1013 | 0 | 20 | -12 | 4.21 |
| Subcallosal Cortex | L | 25 |  | -5 | 11 | -12 | 3.52 |
| Medial Frontal Cortex | L | 11 |  | -5 | 29 | -12 | 3.26 |
| Superior Temporal Cortex | R | 41 | 940 | 50 | -33 | 17 | 3.81 |
| Insula | R | 13 |  | 47 | -25 | 20 | 3.77 |
| Insula | R | 13 |  | 39 | -12 | 17 | 3.62 |
| C: 3-back>2-back: SZ low > HC | | | | | | | |
| Parahippocampal Cortex | L | 19 | 67644 | 33 | -40 | -4 | 5.42 |
| Cerebellum | L |  |  | 8 | -42 | -45 | 5.31 |
| Insula | L | 13 |  | 35 | -40 | 16 | 5.29 |
| Tempral Cortex | L | 38 |  | -48 | 5 | -24 | 5.07 |
| Caudate Nucleus | L |  |  | -5 | 8 | -3 | 3.66 |
| Supplementary Motor Area | L | 6 |  | -5 | -13 | 56 | 3.64 |
| Posterior Cingulate Cortex | L | 31 |  | -8 | -43 | 38 | 3.51 |

*Maxima of regions showing significant BOLD activation differences when comparing all healthy controls and patient sub-groups in the 2-back and 3-back conditions at the whole‐brain level (cluster‐level p<0.05, FDR corr.); Abbreviations used in the text: posterior cingulate cortex – PCC, precuneus – prec, anterior insula – aIN, (pre-)supplementary motor area – (pre-)SMA, temporal cortex – TCx, cerebellum – Cereb, hippocampus – HIPP, caudate nucleus - Caud.*
